# Validation of the 4AT tool for delirium assessment in specialist palliative care settings: protocol of a prospective diagnostic test accuracy study [version 1; peer review: 2 approved]

**DOI:** 10.12688/amrcopenres.12973.1

**Published:** 2021-04-26

**Authors:** Elizabeth Arnold, Anne M. Finucane, Juliet A. Spiller, Zoë Tieges, Alasdair M.J. MacLullich

**Affiliations:** 1 Marie Curie Hospice Edinburgh, 45 Frogston Road West, Edinburgh, Scotland, EH10 7DR, UK; 2 Edinburgh Delirium Research Group, Geriatric Medicine, Usher Institute, University of Edinburgh, Royal Infirmary of Edinburgh, 51 Little France Crescent, Edinburgh, Scotland, EH16 4SA, UK

**Keywords:** 4AT, 4 'A’s Test, delirium, screening, detection, hospice, specialist palliative care, palliative care, DSM-5

## Abstract

**Background:**

Delirium is a serious and distressing neuropsychiatric condition, which is prevalent across all palliative care settings. Hypoactive delirium is particularly common, but difficult to recognize, partly due to overlapping symptoms with depression and dementia. Delirium screening tools can lead to earlier identification and hence better management of patients. The 4AT (4 ‘A’s Test) is a brief tool for delirium detection, designed for use in clinical practice. It has been validated in 17 studies in over 3,700 patients. The test is currently used in specialist palliative care units, but has not been validated in this setting. The aim of the study is to determine the diagnostic accuracy of the 4AT for delirium detection against a reference standard, in hospice inpatients.

**Methods:**

240 participants will be recruited from the inpatient units of two hospices in Scotland. If a patient lacks capacity to consent, agreement will be sought from a legal proxy. Each participant will complete the 4AT and a reference standard assessment based on the diagnostic delirium criteria in the fifth edition of the Diagnostic and Statistical Manual of Mental Disorders (DSM-5). This will be supplemented by tests of cognition and attention, including reverse days of the week, counting down from 20 to 1, Vigilance 'A', the Observational Scale for Level of Arousal, the modified Richmond Agitation Sedation Scale and the Delirium Rating Scale-Revised-98. The assessments will be conducted in a randomized order by two independent clinicians, who will be blinded to the results until both are complete. Primary outcomes will be the sensitivity and specificity of the 4AT in detecting delirium.

**Discussion:**

The findings will inform clinical practice regarding delirium assessment in palliative care settings.

**Trial registration:**

ISRCTN ISRCTN97417474 (21/02/2020).

## Abbreviations

DSM-5: Diagnostic and Statistical Manual of Mental Disorders, 5^th^ edition
DSM-IV: Diagnostic and Statistical Manual of Mental Disorders, 4^th^ edition
DSM-III: Diagnostic and Statistical Manual of Mental Disorders, 3^rd^ edition, Revised
CAM: Confusion Assessment Method
bCAM: Brief Confusion Assessment Method
4AT: 4 ‘A’s test
SQiD: Single Question in Delirium
NuDESC: Nursing Delirium Screening Scale
DOS: Delirium Observation Scale
DRS: Delirium Rating Scale
DRS-R-98: Delirium Rating Scale Revised 98
MDAS: Memorial Delirium Assessment Scale
AMT-4: Abbreviated Mental Test -4
CCC: Concordance Correlation Coefficient
ACCORD: Academic and Clinical Central Office for Research and Development

## Background

Delirium is a serious and distressing neuropsychiatric condition, characterized by an acute disturbance in attention, awareness and cognition. Delirium severity may fluctuate throughout the day, and be associated with disturbances in the sleep-wake cycle. Cognitive changes may impact memory, orientation, language, visuospatial ability and perception^1^. Delirium can be highly distressing for patients and their families and is associated with poor outcomes^2–6^.

Delirium is extremely common across all palliative care settings^7,8^. A recent systematic review estimated the median (range) point prevalence of delirium on admission to palliative care inpatient settings as 32% (6.6%–73%) and period prevalence prior to death as 75% (58%–88%)^7^. Hypoactive delirium is more common than other subtypes in palliative care^9–11^ but may be less noticeable and go under-recognised due to overlapping symptoms with depression, dementia and fatigue^11–13^.

International guidance recommends routine assessment for risk factors of delirium on admission to hospital and other care settings, and subsequently if there are any fluctuations or changes in behaviour or cognition^14–18^. Delirium assessment tools are recommended and may lead to earlier detection. Improved detection may lead to better management, including investigation and treatment of the underlying cause and/or better symptom control, resulting in more favourable outcomes for both patients and their families.

Delirium tools are available for different purposes^19^:

- for delirium detection at first presentation, and at other times when delirium is first suspected: the Confusion Assessment Method (CAM)^20^, shorter versions, including the brief Confusion Assessment Method (bCAM)^21^, the 4 ‘A’s test (4AT)^22^ and informant screening tools, such as the Single Question in Delirium (SQiD)^23^.
- for monitoring of new onset delirium in inpatients, on a regular basis, daily or more frequently: the Nursing Delirium Screening Scale (NuDESC)^24^ and Delirium Observation Scale (DOS)^25^.
- as research assessments: The Delirium Rating Scale (DRS) and the Delirium Rating Scale Revised-98 (DRS-R-98)^26^.

Table 1 shows examples of validation studies in palliative care and oncology populations.

The DRS-R-98, a clinician-rated 16-item scale with 3 diagnostic and 13 severity scales, is able to distinguish reliably from depression and dementia^26^. Use in clinical practice is limited because of training required prior to use and time taken to administer (at least 20–30 minutes with the patient, plus additional time to gather informant history and review case notes)^11,27^.

The Memorial Delirium Assessment Scale (MDAS), a 10-item scale, used for delirium detection and severity monitoring, takes approximately 10 minutes with the patient, plus additional time to gather informant history^30^. It has a simpler format and is easier to rate than the DRS-R-98, but still requires moderate training to administe^r27^. A study in a palliative care inpatient unit demonstrated 97% sensitivity and 95% specificity (cutoff score = 7)^31^.

The CAM and its shorter variants are quicker to administer than either the MDAS or DRS-R-98 (approximately 5–10 minutes), but still require moderate training prior to use, as well as additional cognitive testing^27^. Aspects requiring subjective judgement by the assessor can be more complex and time-consuming to rate, plus there is limited advice for scoring non-verbalizing patients^39^. This may account for high levels of sensitivity and specificity in some studies^33,34^, but lower results when scored by those less experienced^23,33^.

The informant tool, the Single Question in Delirium (SQiD), which asks ‘Do you feel that (patient’s name) has been more confused lately?’, has been validated in oncology patients (80% sensitivity and 71% specificity), but not in palliative care patients^23^. Use of the SQiD is limited when an informant is unavailable.

The 4‘A’s Test (4AT) is a short bedside test for detecting delirium, for use in clinical practice^22^. It takes a few minutes to complete and incorporates 4 items: (1.) An observational measure of alertness, (2.) The Abbreviated Mental Test-4 (AMT4), (3.) The Months of the Year Backwards test, and (4.) Evidence of significant change or fluctuation in alertness, cognition or other mental function arising over the last 2 weeks and still evident in the last 24 hours. The first 3 items are assessed at the patient’s bedside. The last item is derived directly by the assessor or from informant history (i.e. taken from case notes, clinical staff or a relative or carer). The 4AT can be used by any healthcare professional, either at first contact or when delirium is suspected. It is one of the most frequently validated delirium tools in the literature. A recent systematic review and meta-analysis of 17 studies involving 3702 older adults in medicine, surgery, emergency and care home settings, demonstrated a pooled sensitivity of 88% and pooled specificity of 88%^40^. The 4AT’s advantages over other tools are that no special training is required prior to use, and it is quick and easy to administer. Furthermore, all patients can be assessed, including those unable to communicate (patients with severe drowsiness or agitation.)

The 4AT is recommended for identifying patients with delirium^14^, and is currently used in palliative care settings^41–43^, but has not been validated for delirium screening in a terminally ill population.

## Study objective

The study objective is to determine whether the 4AT is a valid tool for delirium detection in specialist palliative care settings. The diagnostic accuracy of the 4AT will be compared against a reference standard delirium assessment, based on the diagnostic criteria of the fifth edition of the Diagnostic and Statistical Manual of Mental Disorders (DSM-5) (Table 2)^1^.

## Methods

### Study overview

**Figure 1:**
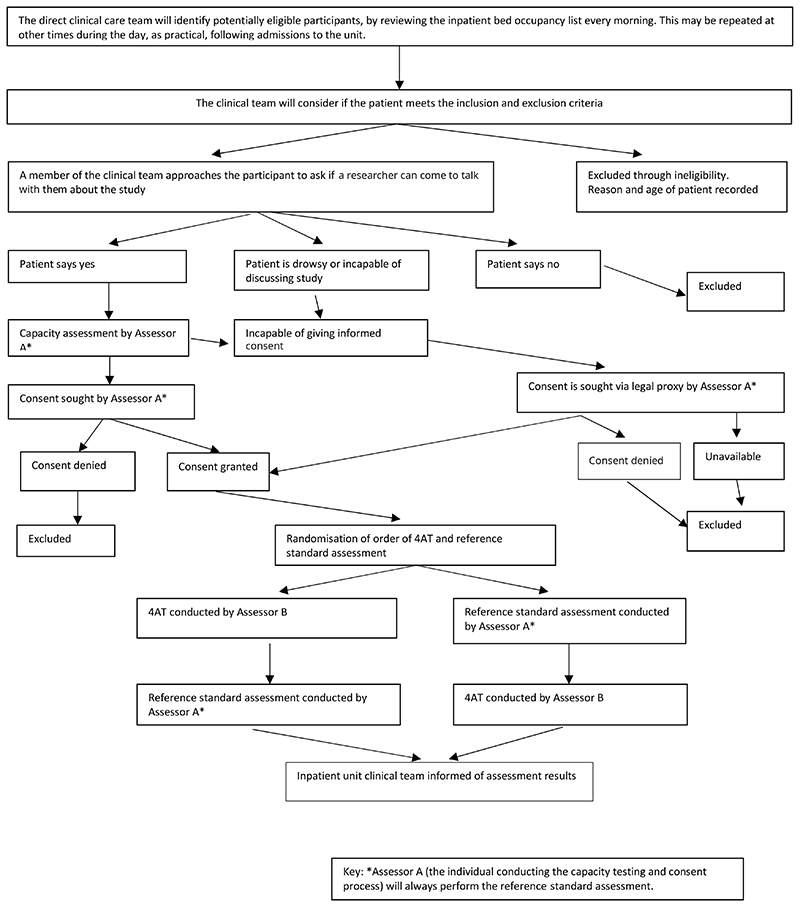
Study overview flow chart (adapted from Shenkin *et al.* (2008)^39^).

In developing this protocol, we drew on the study design and procedures of a previous 4AT validation study conducted in emergency departments and acute medical wards^39^. This study is a prospective diagnostic accuracy study of the 4AT in a representative sample of hospice inpatients. Each participant will independently complete the 4AT and the reference standard delirium assessment based on DSM-5 criteria. Study procedures are described in Figure 1.

### Setting

The study will take place at two hospices in Scotland. The hospices have 17 and 24 inpatient beds, respectively, and admitted a combined total of 700 patients in 2019. Patients with advanced progressive or incurable disease are admitted for complex end of life care, or because they have uncontrolled pain and/or other complex physical or psychological issues, that cannot be managed in other care settings.

### Participants and sample size

Participants admitted acutely to the hospices will be included if they are aged 18 years or over. Patients will be excluded if they are comatosed, unable to communicate in English, are severely dysphasic or have a combined severe hearing and visual impairment, which would limit participation in the study’s tests. Patients will also be excluded if there is a high level of patient and family distress, as judged by the clinical team, or the patient has an acute life-threatening illness requiring time-critical intervention (e.g. suspected spinal cord compression).

A recent meta-analysis of 17 studies on the diagnostic accuracy of the 4AT in a variety of clinical settings, reported a pooled sensitivity of 0.88 (95% CI 0.80-0.93) and a pooled specificity of 0.88 (95% CI 0.82-0.92)^40^. In line with guidance described by Flahault *et al.* (2005)^44^ which suggests a sample size of 176, given a sensitivity/specificity of 0.85, and a minimal acceptable lower confidence level of 0.75, we plan to recruit 240 patients from the inpatient units across both hospices. We estimate that 80% of those recruited will complete the assessments, allowing data analysis on at least 200, slightly above the 176 needed.

### Screening

Eligibility screening will take place every morning, and opportunistically, if practical, throughout the working day, as new patients are admitted to the hospice inpatient units. The clinical care team will identify potentially eligible participants by considering a checklist with the inclusion and exclusion criteria, as shown in Table 3. For patients screened as ineligible, age and reason for ineligibility will be recorded. If the reason for not approaching the patient later resolves (e.g. high level of patient and/or family distress or acute life-threatening illness requiring time-critical intervention), the patient may be approached on another occasion.

### Recruitment processes for participants with and without capacity

Eligible patients will be approached by the clinical team and asked if they would like to hear more about the study. If agreeable, the clinical researcher will provide verbal and written information about the study and invite them to take part. As part of this discussion, the patient’s capacity to consent to participation in the study will be assessed. If the patient is assessed as lacking capacity to decide, their legal proxy will be approached. The legal proxy could be their Welfare Attorney, Guardian or nearest relative (this is the order in which they will be approached). They will be asked to consider the previously expressed views of the patient, and if they think the patient would have wanted to participate in the study. The Adults with Incapacity (Scotland) Act 2000 permits a legal proxy to consent on behalf of an adult with incapacity^45^.

Due to the nature of delirium, it is possible the participant’s capacity to consent may fluctuate. If participants with capacity continue with the assessments shortly after giving consent, they are unlikely to lose capacity within this short space of time. However, if there is a longer period between the capacity/consent process and assessments, it is possible that a participant’s capacity may be lost or regained in the intervening period. The researcher will need to ensure that the capacity and consent remain valid just prior to the assessments being completed. That is, if a participant later regains their capacity after proxy agreement has been obtained, they will be given an opportunity to provide informed consent for themselves. However, if the researcher becomes aware the previously competent participant has now lost capacity, the assessments will continue in view of their previous consent.

Participants are free to withdraw from the study at any time, or the researcher may withdraw a participant. The reason for their withdrawal will be documented, if available, and data collected up to that point, will be used.

### Training of data collectors

The 4AT assessors are hospice inpatient nurses and doctors, who completed delirium training, either during preparation for the study and/or as part of their professional training. Prior to the study, the 4AT was routinely used on admission to the inpatient units, and at other times when delirium was suspected.

The reference standard assessors are also part of the clinical team. Prior to study recruitment, these assessors completed additional training in capacity assessment, obtaining consent and delirium assessment with a Post-Doctoral Research Fellow and psychologist (ZT) and the Chief Investigator (AMJM), to ensure competence. Good Clinical Practice was also completed.

### Assessments

#### 1. Index test

The 4AT takes a few minutes to complete and is described earlier in this protocol^22^.

#### 2. Reference standard assessment

The reference standard assessment may take up to 20 minutes to complete and is based on the delirium diagnostic criteria in the fifth edition of the Diagnostic and Statistical Manual of Mental Disorders (DSM-5) (Table 2)^1^. The battery of tests assessing cognition and attention includes reverse days of the week, counting down from 20 to 1, Vigilance ‘A’^49^, the Observational Scale for Level of Arousal^47^, the modified Richmond Agitation Sedation Scale (the term ‘drowsy’ is used instead of ‘sedation’)^48^ and the Delirium Rating Scale-Revised-98 (DRS R-98)^26^. A diagnosis of dementia or learning disability will be recorded. The assessor will review the patient’s clinical records and speak with someone who knows the patient well, such as a member of the clinical team and/or relatives (with the patient’s consent).

Following the reference standard assessment, the participant will be grouped into one of four categories – delirium present, possible delirium, no delirium, or undetermined, as defined in another recent delirium study^49^ (Table 4).

Where there is uncertainty of the patient’s categorisation, the reference standard assessor will discuss these ‘challenging cases’ with an expert panel. This panel includes a Consultant in Palliative Medicine (JS) and the Chief Investigator, who is a Consultant in Medicine of the Elderly (AMJM). The panel will be blinded to the results of the 4AT, until the final categorisation of the reference standard assessment is complete.

### Data collection

Each participant will complete the 4AT and a reference standard delirium assessment by two independent clinicians. The assessor, who conducted the capacity and consenting process, will always administer the reference standard assessment. Given the fluctuating nature of delirium, the two assessments will be completed within a maximum three-hour time period (target interval 15 minutes). During this period, there will be no communication about the participant between the two assessors, until both assessments are complete (other than to arrange the timing of assessments).

The order of the two assessments will be randomised in a 1:1 ratio. The randomisation allocation will occur immediately following recruitment. An administrator at each site, who is independent of the assessments, will use a block randomised list to direct the order of the assessments.

Once both assessments are complete, the outcome of the reference standard assessment will be communicated verbally to the clinical team looking after the patient, in accordance with the consent process. This is because a provisional research diagnosis of delirium may result in improved patient care.

### Data recording, storage and monitoring

Data will be recorded on paper case report forms and kept securely in locked cabinets, in offices with limited access. Study data collection forms (4AT and reference standard assessment results etc.) will be identified by a participant’s unique identification (ID) number only, to maintain confidentiality. Records with the participant’s name and other personal identifiers (eligibility and recruitment logs, consent forms etc.) will be stored separately from data collection forms with the participant’s ID only.

Clinical researchers will transcribe the data on case report forms into secure databases within Marie Curie Cancer Care IT networks in Edinburgh and Glasgow. The databases will only be accessible to clinical researchers and the Research steering committee, who will be responsible for monitoring the data quality.

### Data analysis

The diagnostic test accuracy of the 4AT for delirium detection in specialist palliative care inpatient populations, versus the reference standard, will be determined using sensitivity, specificity, and positive and negative predictive values. The exact binomial 95% confidence interval will be reported for each measure. A ROC curve analysis will be performed, and the area under the ROC curve and its 95% confidence interval will be reported. Analyses will be completed using IBM SPSS Statistics.

Participants with missing data from the 4AT and reference standard assessments will be included in the statistical analysis, if there is enough information to decide on categorisation. Cases with insufficient information will be excluded, and expert panel advice will be sought where there is uncertainty.

### Data protection

Data will be collected, stored and handled in accordance with guidance from Marie Curie Research Governance committee, the sponsor ACCORD (the Academic and Clinical Central Office for Research and Development) and the NHS Scotland A Research Ethics Committee.

The principle investigator will have direct access to their own site’s data, and to the other site’s on request. To ensure confidentiality, the Research steering committee will only have access to data that has had identifiable participant information removed.

Personal data will be stored securely for a maximum of three years to allow full analysis of the data. Study documentation will not be destroyed without permission from the sponsor.

### Study oversight

There is a process for reporting adverse events, however this is a relatively ‘low risk’ study - the assessors are trained palliative care nurses and doctors, and the study tests are already used in clinical practice. The sponsor, ACCORD, will be responsible for external oversight, which may involve monitoring and audit of study activity and documentation.

### Study status

Recruitment commenced in October 2019. We initially anticipated that recruitment would run for up to 15 months, however recruitment was temporarily stopped in March 2020 due to the coronavirus disease 2019 (COVID-19) pandemic. Recruitment will resume as soon as permitted, in line with research and clinical governance requirements.

### Dissemination of information

The anonymised data will be shared on the ISRCTN Registry within 18 months of trial completion, and we hope to publish the results in an open access peer reviewed journal. The trial was registered retrospectively with ISRCTN on 21^st^ February 2020 (ISRCTN97417474).

### Reporting guidelines

This protocol is reported in line with the SPIRIT guidelines^50^. A completed SPIRIT checklist for this protocol is available on the ISRCTN registry (ISRCTN97417474).

## Discussion/implications

A recent survey of UK palliative care specialists reported the majority (68%) only screened for delirium when suspected clinically, and few (5%) screened routinely on admission to palliative care units^43^. Only a third of respondents (37%) used assessment tools to screen for delirium, despite international guidance advocating their use. Inadequate delirium training and guidance, as well as the ‘complexity of patient’s conditions’ were perceived as barriers to delirium screening. The consequence of health care professionals not using assessment tools, is that delirium, particularly the hypoactive subtype, might go unrecognised and hence untreated in these patients.

The 4AT is a delirium assessment tool with strong evidence of validity in hospitalised patients. Whilst it is used in specialist palliative care settings, it has not been validated. If this study shows the 4AT to be an effective tool in this population, it may be more readily adopted into routine practice than other more complex and lengthy tools. If delirium is detected earlier, it may lead to better outcomes for these patients and their families. The findings from this study will provide evidence for the use of the 4AT in hospice settings, and help inform clinical guidelines and practice in relation to delirium assessment in palliative care.

## Figures and Tables

**Table 1:** Validation studies of Delirium assessment tools used in palliative care and oncology populations^27–29^.

Assessment tool	Validation studies	Study description	Results	Authors’ conclusions
Memorial Delirium Assessment Scale (MDAS)^30^	**33** Hospitalised cancer and AIDS patients (Breitbart, 1997)^30^	MDAS compared to DSM-IIIR and DSM-IV delirium diagnosis	Sensitivity 0.71, Specificity 0.94 (cut-off score 13)	The MDAS is a brief, reliable tool for assessing delirium severity among medical inpatients. It may also be useful in delirium diagnosis.
	**51** Hospitalised cancer and AIDS patients (Breitbart, 1997)^30^	MDAS compared to Delirium Rating Scale (DRS) and Clinician’s Global Rating of Delirium Severity	There was high correlation between MDAS scores and the alternative measures of delirium: DRS (r = 0.88, p < 0.0001) and Clinician’s Global Rating of Delirium Severity (r = 0.89, P < 0.0001).	
	**104** palliative care inpatients (advanced cancer) underwent **330** assessments (Lawlor, 2000)^31^	MDAS compared to DSM-IV diagnosis	Sensitivity 0.97, Specificity 0.95 (cutoff score 7)	The MDAS is a valid and reliable tool for delirium diagnosis and severity monitoring. Proration of MDAS total scores was required in approximately a fifth of delirious patients. This may limit the use of MDAS in research, but be useful in clinical practice.
	**77** palliative care unit inpatients (O’Sullivan, 2015)^32^	MDAS compared to DRS-R-98 in patients with DSM-IV diagnosed delirium	Concordance correlation coefficient (CCC)=0.70	There was substantial overall agreement in the severity scores of the MDAS and DRS-R-98 in palliative care inpatients.
Confusion Assessment Method (CAM)^20^	Pilot study with 32 patients, followed by main study with **52** palliative care unit inpatients (Ryan, 2009)^33^	CAM compared to DSM-IV diagnosis	Pilot study: Sensitivity 0.5, specificity 1.0. Main study (assessors received more training of CAM than in pilot study): Sensitivity 0.88, Specificity 1.0	The CAM is a valid screening tool in palliative care inpatients, but accuracy depends on assessors’ training in its use.
	**21** oncology inpatients (Sands, 2010)^23^	CAM compared to Psychiatrist interview	Sensitivity 0.4, Specificity 0.92.	Primary focus of this study was the SQiD. The CAM performed poorly, likely because the assessors had limited training in its use.
Brief/short Confusion Assessment Method^21^	**51** palliative care inpatients (Rainsford, 2014)^11^	Short CAM compared to DRS-R-98 and clinical judgement	Incidence of delirium was 29% based on clinical judgement alone, but increased to 43% when validated assessment tools (CAM and/or DRS-R-98) were used.	The study supports the short CAM as an appropriate screening tool. The DRS-R-98 is limited as a screening tool by its complexity and time taken to administer.
	**36** palliative care unit inpatients or other inpatients reviewed by palliative care team (Wilson, 2019)^34^	Brief CAM compared to DSM-5 diagnosis	Sensitivity 0.80, Specificity 0.87	The brief CAM was found to have good sensitivity and specificity in veteran palliative care inpatients, but further validation studies with larger sample size are needed.
Nursing Delirium Screening Scale (Nu-DESC)^24^	**59** assessments of 52 haemato-oncology and internal medicine inpatients (Gaudreau, 2005)^24^	Nu-DESC compared to CAM	Sensitivity 0.86, Specificity 0.87	The Nu-DESC, a brief, easy to use tool, demonstrated high diagnostic accuracy in oncology inpatients, but further studies with larger sample size are required.
	**43** palliative care unit inpatients, (Barnes, 2019)^35^	Nu-DESC compared to MDAS	There was positive correlation between the 24 hour maximum and mean Nu-DESC scores and the MDAS (r=0.41, p=0.006, and r=0.40, p=0.008 respectively); but lower or insignificant correlation when Nu-DESC scores using items 2-4 were used.	The Nu-DESC may be useful to monitor delirium severity in palliative care inpatients.
Delirium Observation Screening Scale (DOS)^25^	**48** palliative care unit inpatients (Detoyer, 2014)^36^	DOS compared to CAM	Sensitivity 0.82, Specificity 0.96	The DOS can be used as a screening tool in ‘verbally active’ palliative care inpatients. The tool is quick and easy to score. Further validation studies are required.
	**78** assessments of home hospice patients (Jorgensen, 2016)^37^	DOS compared to DRS-R-98	Sensitivity 0.97, Specificity 0.89	The DOS is a useful observational screening tool in home hospice patients. It is quick to learn and cognitive testing is not required. Further validation studies are required.
	**187** oncology inpatients (Neefjes, 2019)^38^	DOS compared to DRS-R-98	Sensitivity >0.99, Specificity >0.99	The DOS is a brief, accurate screening tool in patients with advanced cancer.
Single Question to identify Delirium (SQiD)^23^	**21** oncology inpatients (Sands, 2010)^23^	SQiD and CAM were compared to Psychiatrist interview	SQiD: Sensitivity 0.8, Specificity 0.71, CAM: Sensitivity 0.4, Specificity 0.92	The SQiD is a quick and easy to use tool, which can be incorporated into the admission process. If the SQiD scores positive, this could trigger a more detailed assessment for delirium. The CAM performed poorly in this study, likely because the assessors had limited training in its use.
4 A’s test (4AT)^22^	None		n/a	n/a
Delirium Rating Scale-revised-98 (DRS-R-98)^26^ and Delirium Rating Scale (DRS)	Used as reference standard delirium assessments in research studies^11,30,32,37,38^			

**Table 2:** Delirium diagnostic criteria of the fifth edition of the Diagnostic and Statistical Manual of Mental Disorders (DSM-5)^1^.

**A.** A disturbance in attention (i.e. reduced ability to direct, focus, sustain, and shift attention) and awareness (reduced orientation to the environment).
**B.** The disturbance develops over a short period of time (usually hours to a few days), represents a change from baseline attention and awareness, and tends to fluctuate in severity during the course of a day.
**C.** An additional disturbance in cognition (e.g. memory deficit, disorientation, language, visuospatial ability, or perception).
**D.** The disturbances in Criteria A and C are not better explained by another pre-existing, established, or evolving neurocognitive disorder and do not occur in the context of a severely reduced level of arousal, such as coma
**E.** There is evidence from the history, physical examination, or laboratory findings that the disturbance is a direct physiological consequence of another medical condition, substance intoxication or withdrawal (i.e. due to a drug of abuse or to a medication), or exposure to a toxin, or is due to multiple etiologies.

Reprinted with permission from the Diagnostic and Statistical Manual of Mental Disorders, Fifth edition (Copyright 2013). American Psychiatric Association. All Rights Reserved.

**Table 3:** Inclusion and exclusion criteria.

**Inclusion criteria**
Aged 18 years or over Acutely admitted to the specialist palliative care inpatient units.
**Exclusion criteria**
Coma Unable to communicate in English (The cognitive tests used have not been validated in non-English language speakers, hence the study only includes patients who can communicate fluently in English). Severe dysphasia Combined severe hearing and visual impairment, which would limit participation in the study’s tests. High level of patient and family distress, as judged by the clinical team. Acute life-threatening illness requiring time-critical intervention (e.g. suspected spinal cord compression)

**Table 4:** Reference standard assessment grouping (adapted from Rutter *et al.* (2018)^49^).

Category	Criteria
Delirium present	All 5 of the DSM-5 delirium core diagnostic criteria are positive
Possible delirium	Some DSM-5 delirium diagnostic criteria are positive (i.e. some features of delirium are present), but not all, due to missing information (perhaps about the history of onset of symptoms).
No delirium present	The core criteria are negative for delirium.
Undetermined	Some, but not all, DSM-5 delirium criteria are positive. This usually represents a resolving or subsyndromal delirium.

## Data Availability

No data are associated with this article.

## Plain language summary

Delirium is a serious and distressing condition, which commonly affects people with terminal illness. Approximately a third of patients will have delirium on admission to hospices. Delirium usually develops over hours to days. It can cause confusion, and problems with memory, concentration, perception (may develop hallucinations) and altered consciousness (people can become agitated or drowsy and withdrawn). It is important to diagnose delirium early, as it increases the person’s chance of recovery. When a person has only hours or days to live, their delirium is less likely reversible, but early detection is still beneficial, as it helps healthcare professionals decide how they can best support the person and their family.

The 4’A’s test or 4AT is a short, easy to administer bedside test, used by healthcare professionals to determine if patients may have delirium. Research has shown the 4AT to be an effective test in hospital patients. It is currently used in hospices, but research is needed to review its use in this setting. This article describes the proposed study to examine the effectiveness of the 4AT in detecting delirium in terminally ill hospice patients.

Patients admitted to two hospices in Scotland will be invited to take part in the study. Those participating in the study will undergo two assessments - the 4AT and another more detailed delirium assessment.

This study will provide evidence about the accuracy of the 4AT in detecting delirium in terminally ill hospice patients. If the study shows the 4AT to be effective, it may be more readily used in routine practice, than other more complex and lengthy delirium tests. If delirium is detected earlier, it may lead to better care for these patients and their families.

## Open Peer Review

**Current Peer Review Status:** ✔ ✔

**Version 1**

Reviewer Report 28 June 2021

https://doi.org/10.21956/amrcopenres.14044.r26708

**© 2021 Bush S.** This is an open access peer review report distributed under the terms of the Creative Commons Attribution License, which permits unrestricted use, distribution, and reproduction in any medium, provided the original work is properly cited.

✔ **Shirley H. Bush**https://orcid.org/0000-0001-8907-1283

^1^ Department of Medicine, Division of Palliative Care, University of Ottawa, Ottawa, ON, Canada

^2^ Bruyère Research Institute, Ottawa, ON, Canada

^3^ Ottawa Hospital Research Institute, Ottawa, ON, Canada

This is a well written study protocol for the validation of the 4AT tool for delirium detection in 2 hospice inpatient units in Scotland. Recruitment for the study commenced in October 2019. The 4AT is a brief 4-item bedside test and is the most commonly used delirium assessment tool in non- intensive care settings in the UK^1^. While the 4AT takes a few minutes to complete and does not require special training prior to its use, the reference standard assessment (based on DSM-5 delirium diagnostic criteria) used in this study may take up to 20 minutes to complete (as includes several tests assessing cognition and attention) and requires assessor training.

Delirium is very common in palliative care patients and remains underdiagnosed, especially the hypoactive psychomotor subtype as palliative care patients commonly experience symptoms such as fatigue and depression. In addition, assessing delirium towards the end of life is often challenging as a patient’s condition declines. This study is warranted as further research is needed in evaluating tools for delirium screening/detection in different palliative care settings and populations^2^.

The study aims to recruit 240 patient participants. I do not have sufficient statistical expertise to be able to comment on the suitability of this sample size.

As this is a study protocol, my response to the peer review question regarding presentation of datasets is 'not applicable', but this was not an available option.

Comments:

The protocol states (in Data Analysis) that “participants with missing data from the 4AT and reference standard assessments will be included in the statistical analysis, if there is sufficient information to decide on categorisation.” The number of patients who are unable to complete particular tests of cognition and attention (done as part of the reference standard assessments)

may provide useful evidence with respect to the utility/ possible burden of these tools in this patient population.

Similarly as this study is being conducted in hospice inpatients, it would be useful to record the Palliative Performance Status, or similar performance status tool, of the patient at the time of the 4AT and reference standard assessments as performance status may predict a patient’s prognosis. I did not see performance status explicitly mentioned in the protocol.

Inclusion and exclusion criteria:

◦ Figure 1: 2nd box: Should it be “patient meets the inclusion or exclusion criteria”?
◦ Table 3: Check language used for inclusion criteria, as a specialist inpatient palliative care unit is usually in an acute care hospital. The term “inpatient hospice” is used in Abstract and Plain Language Summary, so perhaps hospice inpatient unit is a better term here?

Recruitment processes:

◦ Given that trained reference standard assessors need to be available, is the study running seven days a week, or just Monday to Friday?

Training of data collectors:

◦ For clarification within the text, are the 4AT assessors “Assessor B” and are the reference standard assessors “Assessor A” from Figure 1?
◦ Suggest specify how long the 4AT has been in routine use in the 2 hospices as this could impact on how the tool is used in each setting. Has the 4AT been used for a similar or different length of time in each hospice?
◦ Does “Good Clinical Practice” refer to the International Conference on Harmonisation-Good Clinical Practice (ICH-GCP) online training module?

Assessments:

◦ The authors state that “the modified Richmond Agitation-Sedation Scale” would be used, so I had been anticipating the “Modified RASS” as developed by Chester et al.,^3^ but see that the citation is for another modified version of the RASS that was used in older adults in an emergency department.
◦ If a patient is found to be “unassessable” for DSM-5 category 'C', will they also be categorised as “possible delirium” (Table 4)?

Additional minor points:

◦ In original study by Sessler et al. (2002): Richmond Agitation-Sedation Scale was hyphenated ^4^.
◦ In original study by Gaudreau et al. (2005): Nu-DESC was hyphenated (as used in Table 1 of protocol)^5^.
◦ Consider adding the RASS to the list of abbreviations
◦ The MDAS is listed as the 1^st^ tool in Table 1, but is not listed as a delirium tool in the last paragraph of Page 3.
◦ Table 1: There is another validation paper in cancer patients that has recently been published on the SQiD in BMC Cancer^6^.
◦ In data protection paragraph, typo for 'principal' investigator. Suggest add initials here for the PI.

**References**

1. Tieges Z, Lowrey J, MacLullich AMJ: What delirium detection tools are used in routine clinical practice in the United Kingdom? Survey results from 91% of acute healthcare organisations. *Eur Geriatr Med.* 2021. PubMed Abstract | Publisher Full Text
2. Watt CL, Scott M, Webber C, Sikora L, et al.: Delirium screening tools validated in the context of palliative care: A systematic review.*Palliat Med.* **35** (4): 683-696 PubMed Abstract | Publisher Full Text
3. Chester JG, Beth Harrington M, Rudolph JL: Serial administration of a modified Richmond Agitation and Sedation Scale for delirium screening./ *Hosp Med.* **7** (5): 450-3 PubMed Abstract | Publisher Full Text
4. Sessler CN, Gosnell MS, Grap MJ, Brophy GM, et al.: The Richmond Agitation-Sedation Scale: validity and reliability in adult intensive care unit patients./4m */ Respir Crit Care Med.* 2002; **166** (10): 1338-44 PubMed Abstract | Publisher Full Text
5. Gaudreau JD, Gagnon P, Harel F, Roy MA: Impact on delirium detection of using a sensitive instrument integrated into clinical practice.*Gen Hosp Psychiatry.* **27** (3): 194-9 PubMed Abstract | Publisher Full Text
6. Sands MB, Sharma S, Carpenter L, Hartshorn A, et *al.BMC Cancer.* 2021; **21** (1): 75 PubMed Abstract | Publisher Full Text

**Is the rationale for, and objectives of, the study clearly described?**

Yes

**Is the study design appropriate for the research question?**

Yes

**Are sufficient details of the methods provided to allow replication by others?**

Yes

**Are the datasets clearly presented in a useable and accessible format?**

No

***Reviewer Expertise:*** Delirium management and research in palliative care patients; delirium guidelines

**I confirm that I have read this submission and believe that I have an appropriate level of expertise to confirm that it is of an acceptable scientific standard**.

Reviewer Report 22 June 2021

https://doi.org/10.21956/amrcopenres.14044.r26721

**© 2021 Klein C et al.** This is an open access peer review report distributed under the terms of the Creative Commons Attribution License, which permits unrestricted use, distribution, and reproduction in any medium, provided the original work is properly cited.

✔ **Carsten Klein**https://orcid.org/0000-0001-8583-065X

Department of Palliative Medicine, University Hospital Erlangen, Friedrich-Alexander-Universitat Erlangen-Nurnberg, Erlangen, Germany

**Maria Heckel**

Department of Palliative Medicine, University Hospital Erlangen, Friedrich-Alexander-Universitat Erlangen-Nurnberg, Erlangen, Germany

The authors present the protocol of a validation study for the 4AT delirium screening tool for patients treated in specialist palliative care institutions. This tool will be compared to the clinical standard diagnostic procedure for delirium according to the Diagnostic and Statistical Manual of Mental Disorders (DSM 5). The protocol covers an important and highly debated topic, as delirium is common in palliative care and often underdiagnosed. The article is well written and the methods are clearly described. The estimation of the necessary sample size can not be verified by the reviewer, but the parameters chosen appear to reflect the well-known difficulty of achieving adequate sample sizes in palliative care. Inclusion and exclusion criteria are clearly stated. The authors report that assessors for the 4AT will receive training on delirium detection. It would be desirable to know a bit more about the intensity of this training, as the 4AT tool is supposed to be usable without training. Extensive training of staff on delirium detection could be a potential confounder.

Just a minor suggestion: it would be helpful for the reader to have the details of the 4-A-Test readily available. The link to the website (ref. 22) is easily overread, perhaps a sentence about the website and the link could be a service.

Monitoring and reporting recommendations are met and ethical approval is obtained. The consequences of the diagnosis of delirium for the patients are described so that patients receive adequate treatment.

As the authors state, there are already some screening tools for delirium that are validated for use in palliative care settings, each with certain advantages and disadvantages (for a recent review see Watt et al.^1^). The 4AT already has proven usability in different areas and could be of value because of its brevity especially in palliative care.

In summary, this is a well-designed study protocol that hopefully will help to improve screening for delirium in specialist palliative care services.

**References**

1. Watt CL, Scott M, Webber C, Sikora L, et al.: Delirium screening tools validated in the context of palliative care: A systematic review.*Palliat Med.* **35** (4): 683-696 PubMed Abstract | Publisher Full Text

**Is the rationale for, and objectives of, the study clearly described?**

**Is the study design appropriate for the research question?**

Yes

**Are sufficient details of the methods provided to allow replication by others?**

Yes

**Are the datasets clearly presented in a useable and accessible format?**

No

***Reviewer Expertise:*** Palliative Care; Sedation in Palliative Care

**We confirm that we have read this submission and believe that we have an appropriate level of expertise to confirm that it is of an acceptable scientific standard**.
